# Comparing progress toward the millennium development goal for under-five mortality in León and Cuatro Santos, Nicaragua, 1990–2008

**DOI:** 10.1186/1471-2431-14-9

**Published:** 2014-01-15

**Authors:** Wilton Pérez, Leif Eriksson, Elmer Zelaya Blandón, Lars-Åke Persson, Carina Källestål, Rodolfo Peña

**Affiliations:** 1 Department of Women’s and Children’s Health, International Maternal and Child Health (IMCH), Uppsala University, 75185 Uppsala, Sweden; 2 Asociación para el Desarrollo Económico y Social de El Espino (APRODESE), León, Nicaragua; 3 The Centre for Research and Interventions in Health (CIS), León, Nicaragua

**Keywords:** Millennium development goals 4, Equity, HDSS, Nicaragua

## Abstract

**Background:**

Social inequality in child survival hampers the achievement of Millennium Development Goal 4 (MDG4). Monitoring under-five mortality in different social strata may contribute to public health policies that strive to reduce social inequalities. This population-based study examines the trends, causes, and social inequality of mortality before the age of five years in rural and urban areas in Nicaragua.

**Methods:**

The study was conducted in one rural (Cuatro Santos) and one urban/rural area (León) based on data from Health and Demographic Surveillance Systems. We analyzed live births from 1990 to 2005 in the urban/rural area and from 1990 to 2008 in the rural area. The annual average rate reduction (AARR) and social under-five mortality inequality were calculated using the education level of the mother as a proxy for socio-economic position. Causes of child death were based on systematic interviews (verbal autopsy).

**Results:**

Under-five mortality in all areas is declining at a rate sufficient to achieve MDG4 by 2015. Urban León showed greater reduction (AARR = 8.5%) in mortality and inequality than rural León (AARR = 4.5%) or Cuatro Santos (AARR = 5.4%). Social inequality in mortality had increased in rural León and no improvement in survival was observed among mothers who had not completed primary school. However, the poor and remote rural area Cuatro Santos was on track to reach MDG4 with equitable child survival. Most of the deaths in both areas were due to neonatal conditions and infectious diseases.

**Conclusions:**

All rural and urban areas in Nicaragua included in this study were on track to reach MDG4, but social stratification in child survival showed different patterns; unfavorable patterns with increasing inequity in the peri-urban rural zone and a more equitable development in the urban as well as the poor and remote rural area. An equitable progress in child survival may also be accelerated in very poor settings.

## Background

Under-five mortality inequalities hamper the achievement of Millennium Development Goal 4 (MDG4) [1,2]. Since progress toward MDG4 is summarized by national averages, the differences in child survival among socio-economic, regional, gender, and ethnic groups may be overlooked.

Neonatal deaths represent an increasing proportion of mortality in children under the age of five years, in addition to deaths caused by pneumonia, diarrhea, and malaria [3]. Almost all of these deaths occur in low- and middle-income countries (LMIC).

To reach MDG4, countries should maintain an annual average reduction in mortality (AARR) of at least 4.4% [3]. Most LMIC in sub-Saharan Africa have not yet achieved this level and may not reach the goal at the current pace. However reducing under-five mortality inequalities is feasible and can accelerate progress towards MDG4 [4].

There are great variations in child survival within and among LMIC [5]. Ghana is one of the countries in sub-Saharan Africa that has shown rapid progress towards reaching MDG4 [6]. A regional analysis in Brazil found that, although there were social inequalities in child survival, some of the poorest regions had succeeded in reducing under-five mortality faster than the national average [7].

In Latin America, the under-five mortality rate has declined from 54/1000 to 23/1000 between 1990 and 2010 [3]. Although the region is on track towards achieving MDG4, progress has been uneven, a consequence of social inequality among the countries.

Nicaragua is scheduled to reduce under-five mortality by two-thirds between 1990 and 2015 [8]. Our previous study using data from a Health and Demographic Surveillance System (HDSS) showed that León is improving in child mortality combined with increasing social equity in survival [9]. National surveys have indicated that the AARR among the seventeen Nicaraguan departments that constitute the country’s administrative-political territory ranged from -1.4% to 15% between 1998 and 2006 (Author’s calculation based on DHS data) [10]. If these rates continue, MDG4 may not be reached by two-fifths of the departments. Our aim was to examine under-five mortality trends with regard to social and regional inequalities in two areas where population-based data is available from HDSS.

## Methods

### Study setting

The municipality of León and the Cuatro Santos area are located in the Nicaraguan Pacific region. León is 93 kilometers and Cuatro Santos 250 kilometers from the capital, Managua. León is 80% urban and has a population of 172,000. Cuatro Santos is a rural area divided into four municipalities with a total population of 25,000. Agriculture and animal husbandry predominate in rural areas, while a labor market characterizes the urban areas. In 2002 of 132 municipalities, the human development index averaged 0.745 in León (rank number 124), compared to 0.524 in Cuatro Santos (rank number 27) [11].

### Health services

The Nicaraguan health system includes public and private services [12]. The former consists of hospitals, health centers with general practitioners and nurses, and smaller health centers. Hospitals also have specialists on their staff. Private clinics are found only in the cities and their services are sold to the public or contracted by the Nicaraguan Social Security Institute. The municipality of León contains a teaching hospital, three main health centers, and 23 smaller health centers. Cuatro Santos has four larger health centers and nine smaller health centers, with the closest hospital 130 kilometers away.

### Surveillance site description and study design

The León HDSS includes a baseline survey performed from 2002 to 2003, which contained a sample of 55,000 inhabitants (20% residing in rural areas). This was updated once in 2004 and twice in 2005. More information on the León HDSS can be found elsewhere [9,13]. The rural Cuatro Santos HDSS was established with a baseline survey in 2004, followed by updates in 2007 and 2009. It consists of 5,000 households. In Cuatro Santos the HDSS covered 100% of the study population. Information on vital statistics (i.e., births, deaths, and migration), reproductive histories of women 15–49 years, and data on household characteristics (i.e., water, sanitation, and walls) was collected from these open cohorts in both areas. Both HDSS are only representative of the urban and rural areas of the Pacific region of Nicaragua.

### Data collection

Female interviewers collected data on births and deaths of children under the age of five years by taking histories of women who were of reproductive age (15 to 49 years old). The birth histories of mothers who migrated out from the study area were not updated.

Causes of child deaths were ascertained by means of a standard verbal autopsy interview (VA) based on the World Health Organization and the International Network for the Demographic Evaluation of Populations and Their Health (INDEPTH) recommendations [14]. The generic VA questionnaire was translated and adapted to Spanish, and three physicians in León and two physicians in Cuatro Santos independently interpreted the obtained information in order to ascertain causes of death according to the International Classification of Diseases, 10^th^ edition. The VA surveys were conducted in León in 2009 and in 2010 in Cuatro Santos for all deaths that had occurred after the baseline survey in the two study areas.

### Definitions

The under-five mortality rate (U5MR) was defined as the number of deaths before the reaching the age of five divided by the number of live births for the same time period. The AARR was defined as the percentage of mortality reduction that is reduced on average in one year. Maternal education was categorized as either completed primary school or beyond or not completed primary school (that includes illiterate women and literate women who had not completed primary school) [15]. The fertility rate was defined as children per woman of reproductive age (15 to 49 years).

### Statistical analysis

The annual U5MR was calculated from 1990 and 2005 for León and from 1990 to 2008 for Cuatro Santos. To reduce random variations in the time series, we smoothed the mortality rate trend using a three-year moving average and this average was assigned to the second year.

To compute the AARR, we used the proposed measure average annual percent change (AAPC) taking into account the autocorrelation in the time series. We will use AARR and AAPC interchangeably in this study. It assumes a nonlinear trend of the U5MR over the study period. The AAPC is based-on segmented partitions of the time series and computed as a weighted average of slope coefficients. The AAPC is defined as:

$$\text{AAPC}=\left(\sum_{j=1}^{k+1}\omega_{j}\beta_{j}\right)-1$$

where ‘exp’ is the exponential e = 2.71, ‘ω’ is the weight which is equal to the number of time points within each segment, ‘k + 1’ is the number of segments, and ‘β’ is the slope of the regression line in each segment of time [16]. Progress towards MDG4 was assessed as on track, insufficient, or no progress made. On track indicated that U5MR was < 40 child deaths per 1000 and AAPC at least 4.0%; insufficient was U5MR > 29 deaths per 1000 and AAPC between 0.9% and 4.0%; and no progress was U5MR > 29 deaths per 1000 and AAPC < 0.9% [17,18].

The expected U5MR by 2015 was computed with the formula:

$$U5MR_{2015}=U5MR_{b}*{\left(1–\text{AARR}\right)}^{2015–b}$$

[19] We set a constant AARR at 4.4% to project the expected U5MR by 2015, using 1990 as the base year. Furthermore, we projected the U5MR_2015_ replacing the AARR with the observed AAPC and taking the last year of the smoothed mortality trend as the base year. The number of child deaths and cause-specific fractions were measured by cause and setting. Pearson or Fisher Chi-square was analyzed and *p* < 0.05 considered significant.

Social inequality was assessed comparing the U5MR by maternal education levels in León (urban and rural) and Cuatro Santos. The mortality ratio and mortality differences with 95% confidence interval were calculated for this purpose. The category of references was the child mortality in mothers with completed primary education or more. Furthermore, we performed a Cox regression with robust standard errors between the level of education of the neighborhood relative to the level of education of the mother and under-five mortality for three time periods: 1990 to 1994, 1995 to 1999, and 2000 to 2005. The model was adjusted for maternal age, parity, and study setting (urban León, rural León, and Cuatro Santos). The hazards ratio with its respective 95% confidence interval represented the mortality gap between the two levels of maternal education. Analyses were performed in Stata 12.0 (Stata Corporation, College Station, Texas) and the calculation of the average annual percent change was done with the Joinpoint public software regression program 4.0.4 (http://surveillance.cancer.gov/joinpoint/).

### Ethical considerations

The ethics committee at the Autonomous National University in León, Nicaragua, has given its approval to the HDSS and for the use of the data for this study in León and Cuatro Santos as part of a doctoral research project by WP during the period 2008–2012. Permission to use the dataset for this study was obtained from the coordinators of the HDSS in León and Cuatro Santos. Informed verbal consent was obtained from each person interviewed regarding cause of death.

## Results

Table 1 describes demographic and household characteristics for the three settings at baseline. Urban León had the lowest fertility rate, followed by Cuatro Santos and rural León. The proportion of women with completed primary education was higher in urban León than in the two rural settings. However, primary education level and presence of latrines was higher (*p* < 0.05) in rural León than in Cuatro Santos, and Cuatro Santos also showed a higher level of poverty when compared to León.

**Table 1:** Background information at baseline of HDSS in León and Cuatro Santos, Nicaragua

	***León***			***Cuatro Santos***
	***Rural***	***Urban***	***Total***	***Total***
Households (n)	3,273	7,271	10*,*994	4,451
Population (n)	16,412	38,235	54,647	24,535
Women (15–49 years) (n)	4,001	11,215	15,216	5,844
Total fertility rate	3.0	2.0	2.2	2.8
Percentage of women with primary education (15–49 years) (SE^1^)	39.4 (0.77)	82.9 (0.35)	71.4 (0.36)	50.6 (0.65)
Percentage of households with piped drinking water (SE)	15.9 (0.63)	95.5 (0.24)	71.8 (0.42)	18.0 (0.57)
Percentage of households with latrine (SE)	83.0 (0.65)	98.3 (0.15)	93.8 (0.22)	73.4 (0.66)

^1^SE: Standard Errors as percentage.

A total of 24,385 births (32% in rural areas) were recorded in León from 1990 to 2005, and a total of 12,879 births in Cuatro Santos from 1990 to 2008. The number of under-five deaths in urban León, rural León, and Cuatro Santos were 446, 313, and 408, respectively.

**Figure 1:**
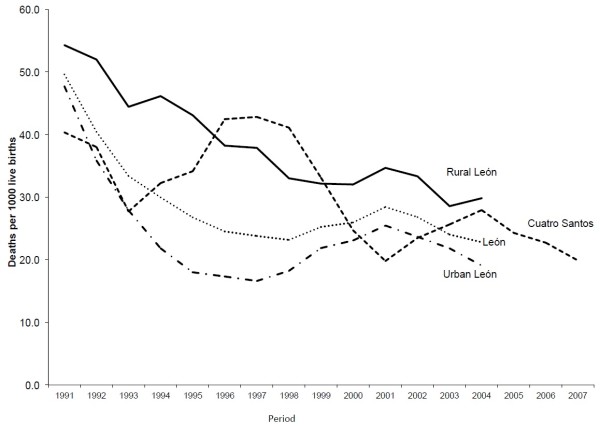
Under-five mortality rate in León and Cuatro Santos, Nicaragua 1990 to 2008 expressed as three-year moving averages.

The U5MR declined in all three settings during the study period (Figure 1). The U5MR declined about twice as much in urban León, as in rural León and Cuatro Santos. Rural León showed an almost linear decline, but urban León had a faster reduction from 1991 to 1995. Then, from 1997 to 2001, the U5MR increased, experiencing another reduction after 2001. Cuatro Santos had more variable trends with ups and downs in different time periods. The initial reduction was during the first three years of the study period, and then it increased until 1997. A reduction was observed for the next four years like in the first period, and finally a short increase was observed between 2001 and 2004 followed by a slow reduction by 2007.

Urban León is the setting with the highest observed AAPC. Between 1990 and 2005, rural León showed a higher AAPC than rural Cuatro Santos (Table 2). The three settings (urban León, rural León, and Cuatro Santos) are on-track to achieve the MDG4 target by 2015. With the observed AAPC urban León may surpass the target; meanwhile the rural settings might reach an expected U5MR sufficient to achieve the MDG4 target with the observed AAPC.

**Table 2:** Progress toward MDG4 in León (urban and rural) and Cuatro Santos from 1990 to 2008

	***Urban León***	***Rural León***	***Cuatro Santos (rural)***
U5MR decline (%)	60.5	45.1	36.4/58.1^1^
AAPC observed (%)	8.5	4.5	3.7/5.4^1^
MDG4 target 2015	16.2	18.4	13.7
Progress toward MDG4	On track	On track	On track
AARR required 2005–2015/2008–2015	1.3	4.3	6.4/4.6^1^
Expected U5MR by 2015 based on observed AAPC	7.2	17.9	12.7

^1^During 1990–2005/ 1990–2008.

Table 3 shows the social inequalities over time and progress towards MDG4 in each setting. In León, 35 mothers did not have primary education (equivalent to 50 live births) between 1990 and 2005. Child mortality was higher among children of mothers without primary education, except in rural León from 1990 to 1994 and rural Cuatro Santos from 2000 to 2008. In urban León AAPC from 1990 to 2005 was higher for mothers without primary education (AAPC = 11.6%) than for those with primary education or more (AAPC = 6.6%). In contrast, the AAPC for the same years in rural León was higher in the group of mothers with completed primary education or more (AAPC = 9.6%), than mothers with lower education level (AAPC = -0.2%). In Cuatro Santos, the AAPC from 1990 to 2008 was similar for both levels of maternal education.

**Table 3:** Under-five mortality rate and annual per cent reduction (AAPC) by maternal education level and time period in León (urban and rural) and Cuatro Santos (rural), Nicaragua, 1990 to 2008

	***Completed primary education or more***					***Less than primary education***				
	***Births***	***Deaths***	***Rate (95%CI)***	***AAPC (%)***	***Births***	***Deaths***	***Rate (95%CI)***	***AAPC (%)***	***Rate ratio (95%CI)***	***Difference (95%CI)***
*Urban-León*										
1990–1994	3,628	101	27.8 (22.5, 33.2)		3,389	176	51.9 (44.5, 59.4)		1.8 (1.4, 2.3)	24.1 (14.7, 33.5)
1995–1999	3,361	45	13.4 (9.5, 17.3)		1,014	34	33.5 (22.5, 44.6)		2.5 (1.6, 3.9)	20.1 (8.2, 32.0)
2000–2005	4,299	87	20.2 (16.0, 24.4)	6.6	944	32	33.9 (22.4, 45.4)	11.6	1.6 (1.1, 2.5)	13.7 (1.1, 26.1)
Progress				On track				On track		
*Rural-León*										
1990–1994	554	23	41.5 (24.9, 58.1)		2,328	125	53.7 (44.5, 62.9)		1.3 (0.8, 2.1)	12.1 (-7.2, 31.5)
1995–1999	617	16	25.9 (13.4, 38.5)		1,583	63	39.8 (30.2, 49.4)		1.5 (0.8, 2.6)	13.8 (-2.1, 29.9)
2000–2005	1,002	17	17.0 (9.0, 25.0)	9.6	1,616	68	42.1 (32.3, 51.9)	-0.2	6.4 (3.7, 10.9)	57.3 (40.4, 74.2)
Progress				On track				No progress		
*Cuatro Santos*										
1990–1994	1191	34	28.5 (19.1, 38.0)		2380	89	37.4 (29.8, 45.0)		1.3 (0.8, 1.9)	8.8 (-3.2, 21.0)
1995–1999	1268	40	31.5 (21.9, 41.2)		2316	106	45.8 (37.3, 54.3)		1.4 (1.01, 2.0)	14.2 (1.4, 27.0)
2000–2005	1797	40	22.3 (15.4, 29.1)		2202	56	25.4 (18.9, 32.0)		1.1 (0.7, 1.6)	3.2 (-6.3, 12.5)
2006–2008	961	18	18.7 (10.2, 27.3)	3.4/4.8 ^1^	764	17	22.3 (11.8, 32.7)	2.1/5.2^1^	1.2 (0.6, 2.2)	3.5 (-9.9, 17.1)
Progress				On track				On track		

^1^Decline from 1990 to 2005/1990 to 2008.

Between 1990 and 1994, the highest hazard ratio (HR) of mortality occurred among children of mothers without primary education whose neighbors also had a low education level (HR = 1.7, 95% CI: 1.2–2.5), while the lowest risk was among mothers with primary education residing among neighbors with the same education level (HR = 1.1, 95% CI: 0.7–1.7). During the last two time periods, the association of maternal education relative to average education in the neighborhood was not significant (data not shown).

A total of 59 under-five deaths in León and 39 in Cuatro Santos were analyzed to ascertain the cause of death (Table 4). Ten planned interviews were not performed in León (three people declined, and in seven cases an appropriate respondent could not be found); one was not performed in Cuatro Santos (an appropriate respondent was not found). The proportion of neonatal deaths was higher in León (59%) (*p* < 0.05) than in Cuatro Santos (41%). Infectious diseases (diarrhea, pneumonia, and other infections) after the first month of life were responsible for 22% of the deaths in León and 41% in Cuatro Santos.

**Table 4:** Causes of under-five mortality based on verbal autopsy interviews in León (2003–2007) and Cuatro Santos (2004 to 2008), Nicaragua

***Diagnosis***	***León n (%)***	***Cuatro Santos n (%)***
Neonatal deaths	35 (59.3)	16 (41.0)
Diarrhea and gastroenteritis of presumed infectious origin	5 (8.4)	4 (10.3)
Pneumonia	5 (8.4)	7 (17.9)
Other infectious diseases (post-neonatal)	3 (5.1)	5 (12.8)
Congenital malformations	5 (8.4)	4 (10.3)
Injuries (post-neonatal)	2 (3.4)	1 (2.6)
Other causes	4 (6.8)	2 (5.1)
Total	59 (100.0)	39 (100.0)

## Discussion

### Main findings

In three rural and urban Nicaraguan settings overall child survival had improved sufficiently to reach the MDG4 by 2015. There were, however, differences in the rate of progress. It was faster in urban León than in both rural areas surveyed, as well as in comparison with the national level [8]. Using maternal education level as a social characteristic we found that the reduction in mortality was combined with greater equity in survival not only in the wealthier urban area but also in the poorest of the three study areas. Neonatal deaths accounted for a high percentage of under-five mortality, in addition to infectious diseases, indicating a major opportunity for further improvement in child survival by implementing a high-coverage perinatal care in all areas.

### Methodological issues

The possibility of recall bias in birth histories is well documented and may affect mortality estimations. To minimize such bias, local calendars were used during interviews to help the respondent to precisely remember the birthdate and the date of death of their children [9,15]. In León, information on maternal education was obtained in the reproductive survey conducted in 1996 and updated during routine visits to the same study clusters of the HDSS [9,13]. Because the small number of deaths resulted in unstable rates, we operationalized maternal education in two categories. Unfortunately, this did not allow analysis of the inequity gradient of mortality beyond this dichotomy (e.g., incomplete primary education, completed primary school, incomplete secondary education, and completed secondary education and above). In Cuatro Santos this information was measured from the HDSS baseline (2004) and onwards covering the situation from 1990 in the stratified analysis of child mortality.

Poverty is another measure applied in studies on inequities. Both HDSSs use unsatisfied basic needs to assess poverty [9,15]. However, for those births that occurred ten years before baseline, this measure of poverty might be unreliable. The number of deaths was small at the end of the study period (mainly in Cuatro Santos) limiting the statistical power in the multivariate analysis. Non-participation in the data collection was less than 0.1% in both León and Cuatro Santos.

### Trends and equity in child survival

Urban León is on track to reach the MDG4 before 2015. Two different trends were observed in urban Leon. First, the rapid progress between 1991 and 1996 in comparison with rural León may be explained by higher provision of social services (including piped water and sanitation), easier access to health services and more resources in the households, including a higher proportion of mothers with primary or higher education. Evidence from sub-Saharan countries was not consistent with this finding, where demographic dynamics negatively impacted housing conditions, access to healthcare, and child survival [20]. Some evidence suggests that urbanization may improve child survival [21]. Second, between 1997 and 2001, the U5MR increased, a period also characterized with a high inequity gap in child survival between social groups in the urban area. It might reflect the situation of child health in peri-urban blocks where the population lives in a less healthy environment than in rural areas, where migrants from rural areas often settle [22,23]. However, the current data set does not allow a more detailed analysis of urbanization, poverty and child health in urban, peri-urban and rural areas, a relevant settings issue in low- and middle-income countries [24-26].

Although rural León is on track toward MDG4, the almost linear decreasing mortality trend was accompanied with widening inequalities. This scenario is often found in LMICs [27,28], indicating that lifesaving interventions are not reaching the most disadvantaged socioeconomic groups, or their health seeking is delayed [29-31]. One study in Nicaragua reported that the nearest public health service is the one most accessed by the poorest people, but these facilities lack the resources to deal with serious illness. Mothers must incur high costs in order to obtain good quality health care, often by traveling to a city [32].

Baseline mortality rate in Cuatro Santos was lower than in rural and urban León. A hypothesis is that León was one of the zones more affected by the war during the 1980s. The annual trend reveals a cyclical pattern every three or four years in Cuatro Santos. Two possible explanations may be either the presence of a random variation due to small number of child deaths or seasonal patterns. Extreme climate variability raises the pluvial level and it is associated with an increase of the incidence of infectious diseases like diarrhea and respiratory diseases, mainly affecting children. For example, outbreaks of rotavirus and leptospirosis have most affected the Pacific region of Nicaragua and likely the response of the primary and curative health services in Cuatro Santos may have been limited, in comparison to León [33,34]. The sudden increase observed between 1994 and 1997 may also be explained by a migration of refugee families that were displaced from the area during the 1980s to neighboring areas in Honduras, where they lived in precarious health conditions. After the war, that population group returned to Cuatro Santos (Elmer Zelaya Blandon, personal communication, September 2013). Studies in African contexts have found higher childhood mortality among former refugee populations in comparison with mothers that never emigrated [35].

Despite these changing trends experienced in Cuatro Santos, the poorest region in our survey, child mortality trends revealed rapid progress to reach the MDG4 combined with greater social equity in child survival over time in comparison with rural León.

This is contrary to the common pattern in low-income societies, although other examples exist of similar patterns to those in Cuatro Santos [27,36]. In this area poverty is widespread but with some decline from 67% in 2004 to 55% in 2009 [37]. Investment has been made in the area, like improved roads that may improve commerce and reduce poverty and there is also improved access to health services. Improvement to the safe drinking water supply to households and sanitation has also taken place, which is essential for child health [38]. This geographical area has experienced substantial emigration that may affect economic development through the influx of remittances. One-fifth of households in Cuatro Santos have at least one family member who has migrated, mainly to Costa Rica, El Salvador, Guatemala, or Honduras [39]. Education is considered a determinant of child survival and reduction of inequalities [40]. Studies in poor settings have found that educated women are more capable of understanding health information, demanding access to healthcare services, and carrying out other health seeking behaviors than less educated women [41].

An individual’s socio-economic position relative to the socio-economic position of the neighborhood may be associated with inequalities in health outcomes [42]. Peña et al. (2000) found that impoverished mothers living in poor neighborhoods experienced lower levels of child mortality compared to poor mothers living in more affluent neighborhoods [15]. The mechanism was reportedly that poor people living among other poor people might have a stronger social support network for providing resources for health than poor people living among non-poor. In our study, in which we used education instead of poverty as a socio-economic indicator, the maternal position in the neighborhood seems to have an important influence on child survival during the early years of the study. This might indicate that mothers copy the health behaviors of other people in the neighborhood, with a worse scenario for child survival when mother and neighborhood have low levels of education. Education is a form of social capital, and mothers with no primary education may not receive appropriate advice or social position to manage severe child illnesses.

### Causes of deaths

In both León and Cuatro Santos neonatal causes dominated among the under-five deaths, followed by infectious diseases, especially in rural Cuatro Santos. This is a pattern found in most LMICs [43]. It should be noted that in spite of a relatively low level of mortality in comparison with other LICs, diarrhea deaths are still a problem, highlighting issues related to water and sanitation as well as access to rehydration therapy. Further analysis of neonatal causes of death may suggest possible preventive strategies within the perinatal health services in the areas.

## Conclusions

The three geographical areas in our study were all on track to reach MDG4, but only two showed improved equity in child survival. The urban area with better health services and more educated mothers but also the remote rural area with only primary health care services and less educated mothers showed this favorable pattern. The rural area surrounding the city of León had sustained social inequality in child survival rates. Our findings show that reduction in mortality before the age of five years can be combined with greater equity in child survival, even in a very poor society.

## Competing interests

The authors declare no competing interests.

## Authors’ contributions

EZ, RP, LÅP, and CK designed the HDSS. LE and EZ performed quality control on the data from the Cuatro Santos HDSS. WP participated in data supervision for the León HDSS. WP did the statistical analysis and wrote the manuscript. LE, EZ, LÅP, CK, and RP shared in interpreting the data. All authors read and approved the final draft of the manuscript.

## Pre-publication history

The pre-publication history for this paper can be accessed here:

http://www.biomedcentral.com/1471-2431/14/9/prepub
